# Burnout Persists in Teachers in Ireland Post-COVID-19: A Qualitative Follow Up Comparative Study

**DOI:** 10.3390/ijerph22040641

**Published:** 2025-04-18

**Authors:** Ellen Ní Chinseallaigh, Matthew Shipsey, Elisha Minihan, Blanaid Gavin, Fiona McNicholas

**Affiliations:** 1 School of Medicine, University College Dublin, Belfield, D04 C1P1 Dublin, Irelandfiona.mcnicholas@ucd.ie (F.M.); 2 Children’s Health Ireland (CHI) at Crumlin, D12 N512 Dublin, Ireland; 3 Lucena Clinic, Saint John of God, Child and Adolescent Mental Health Services, Rathgar, D06 HX93 Dublin, Ireland

**Keywords:** occupational stress, burnout, post COVID, teachers, education, qualitative

## Abstract

Teacher burnout, a combination of emotional exhaustion, depersonalization, and diminished personal accomplishment has been increasing, notably during the COVID-19 pandemic. Our 2022 study revealed significant burnout levels, identifying that teachers’ pandemic experiences had adverse “Consequences” that left them feeling “Overburdened”, and “Abandoned”. Although COVID-19 has receded, recent findings indicate that Irish teachers continue to face heightened demands without adequate support. This follow-up comparative study aims to deepen understanding of post-pandemic teacher burnout by using the same questions from the 2022 study to understand and analyse these evolving stressors. A qualitative, comparative approach was employed. Participants (*n* = 337) were recruited from various school types across Ireland. A Study Specific Questionnaire (SSQ) with open-ended questions allowed for thematic analysis, comparing responses with 2022 themes to explore continuity and changes in burnout experiences. Thematic analysis revealed four major themes: (1) Administrative Overload—increasing paperwork and curriculum changes; (2) Unrealistic Expectations—pressures from parents, society, and authorities; (3) Lack of Community Support and Empathy—teachers reported a sense of isolation and a need for mutual support; and (4) Inadequate Mental Health and Professional Support—insufficient mental health resources for both educators and students. The study underscores the urgent need for systemic changes to address teacher burnout in Ireland. Recommendations include reducing administrative load, clarifying professional boundaries, fostering empathy within the school community, and expanding mental health services. Addressing these factors is essential for sustaining a resilient educational system in the post-pandemic context.

## 1. Introduction

Burnout, a psychological syndrome which emerges as a prolonged response to chronic interpersonal stressors, is characterised by three key dimensions: emotional exhaustion, depersonalization, and a sense of reduced personal accomplishment or efficacy [1] As indicated by its placement and definition within the 11th revision of the International Classification of Diseases (ICD-11), burnout is a syndrome defined by context—namely, it is an occupational phenomenon [2]. As such, the occupational context is a key determinant of burnout prevalence. In other words, not all occupations are equally susceptible to burnout. The teaching profession has for many years been recognised as an at-risk occupation for burnout [3].

Over time, theoretical models have evolved and highlighted the dynamic interplay between work-related demands and resources [4]. The Job Demands–Resources (JD-R) model emphasises the balance between job demands—such as workload, roles and responsibilities and time pressures, and resources, including personal and social support, autonomy, and professional development opportunities [5]. Empirical research supports this model, demonstrating that excessive occupational demands lead to emotional exhaustion, while inadequate resources are aligned to disengagement [6]. Among teachers, studies show that they face substantial job demands, including pedagogical and administrative workload, managing student (and parent) behaviour, and poor physical work environments while often lacking sufficient resources such as collegial support, decision-making autonomy, and appreciation [7].

A large-scale (N = 2035) study of Finnish teachers provided strong empirical support for the JD–R model, demonstrating that job demands predicted ill health through their impact on burnout, while job resources were associated with greater work engagement and organisational commitment. Their findings highlight how burnout mediates the relationship between workplace conditions and both teacher well-being and retention [7]. Other researchers working in Norway have extended the JD-R model by exploring the relationships between different types of job demands and resources, and relationship with different dimensions of burnout. In their study, the specific demand of “Time pressure” was the strongest predictor of emotional exhaustion with no impact on teacher cynicism, while low student motivation and working in a dissonant value context was predictive of cynicism but not the BO dimension of emotional exhaustion [8].

These concerns pre-dated the COVID-19 pandemic. The pandemic exacerbated these issues, introducing new stressors and intensifying existing ones [9]. Post-pandemic research has consolidated the finding that the teaching occupation is particularly at-risk for burnout [10]. The implications of teacher burnout are profound, affecting both educators, students, families, and society at large. For teachers, burnout can lead to decreased job satisfaction, higher absenteeism, and increased rates of attrition. A recent meta-analysis concluded that symptoms of burnout show a significant positive relationship with teachers’ intentions to quit, and that the risk of teacher attrition from burnout may be increasing over time [11]. For students, meanwhile, teacher burnout can result in lower educational outcomes and decreased engagement [12]. As such, burnout among teachers poses a critical threat to the education system.

Teachers in Ireland, like their colleagues worldwide, bore considerable responsibility in the uncertain times of the early pandemic years. Research has shown how Irish teachers felt unsupported in navigating the abrupt transformation of the educational landscape, citing insufficient resources, lack of clear guidelines, and inadequate mental health support systems [13]. Furthermore, teachers struggled with the dual stress of managing their own anxieties about the virus alongside their students’ anxieties. This professional and emotional burden led to significant stress and negatively impacted on teachers’ mental health and well-being [14]. Our team contributed to this field of study with mixed methods research in a national sample of 245 Irish teachers [15]. Using the Copenhagen Burnout Inventory as the primary outcome measure, 79–82% of participants reported moderate or high levels of work and personal burnout, with perceived adverse effects on their physical health and mental health. More than half (58%) had seriously considered changing jobs within the past 6–12 months. Key emergent themes from the qualitative component of this work identified respondents’ sense of being “Overburdened” and “Abandoned” and with adverse “Consequences” on their physical and mental health [16]. Participants described issues such as large class sizes, high workload, and out-of-work contact, along with a sense of being criticised and unappreciated by others [16].

The WHO declared an end to the COVID-19 pandemic in May 2023. In the post-pandemic era, teachers are facing a new set of challenges, including the need to implement health protocols and address the learning gaps that were amplified during remote instruction in a generation with unprecedented rates of poor mental health [17]. Rather than improving, or even returning to pre-pandemic levels, some studies report a decline in teachers’ working conditions in terms of classroom disruptions, student responsibility, and safety concerns [18]. Work by our own group also signalled a deterioration in levels of teacher burnout even following pandemic offset. Rates of burnout increased across all domains; work, personal and student-related between 2020/21 and 2022/23 [19]. Furthermore, teachers reported a lack of organisational stress training or stress reduction activities and ongoing perceptions of decreased job satisfaction and ability.

This growth in teacher burnout signals an urgent need to better understand the multifaceted causes and course of this complex issue. Only with adequate data and a deep understanding of the intricate issues at play in teacher burnout can effective strategies be developed to mitigate its impact.

This follow-up qualitative study aims to provide a comparison of the experiences of occupational stress amongst teachers in Ireland in the post-pandemic era. By examining the impact of the pandemic on teacher burnout and well-being three years post onset, we aim to extend the data to enrich the understanding of this pressing issue.

## 2. Material and Methods

Ethical approval was granted by University College Dublin (LS-22-43-Minihan-McNicholas).

### 2.1. Participants

This study utilised the same recruitment strategy as the original 2022 study for direct comparison. Both teaching staff and principals were invited to participate through completion of a survey, the development of which is described below.

Several recruitment strategies were deployed to maximise response rate. Firstly, schools were stratified to include representation from mainstream primary, special primary and post-primary schools as well as geographical representation from the four provinces of Ireland and school principals nationwide were contacted. These principals were invited to participate and asked to distribute the survey to teachers within their schools. Secondly, individual teachers and principals across the country were invited to participate through the Irish National Teacher’s Organisation (INTO) InTouch magazine and e-newsletter. A follow-up reminder with the survey link was published in the same magazine and e-newsletter towards the end of the data collection period. Thirdly, attendees at the Irish Learning Support Association (ILSA) Conference were provided with a hardcopy of the survey and invited to return completed questionnaires into a return box at the conference.

### 2.2. Questionnaire

The anonymous questionnaire utilised in this study was identical to that used in the original 2022 study to allow direct comparison. The research team developed a Study Specific Questionnaire (SSQ), available in digital and hardcopy formats with three questions offering free text response options. This included the standardised Copenhagen Burnout Inventory (CBI) along with some study specific questions designed to capture the experiences during the pandemic. The three questions were used by the research group in previous research on occupational stress and burnout and were as follows: (1) Have you any suggestions about what you think should change in your work to reduce the risk of burnout? (2) What can your organisation do better to support staff? The third question was an open invitation to “add anything you wish”. Despite the survey including a different teacher cohort compared to the 2022 study, the use of the identical methodology allows for a meaningful comparison of burnout levels as the pandemic continues to affect educators. Quantitative data are reported from this sample elsewhere [20]. This paper reports on the three open-ended questions only.

### 2.3. Analysis Method

All responses were anonymous and analysed collectively. As per the previous study, a qualitative approach employing the Braun and Clarke method of thematic analysis was used. Free text responses were exported and shared to the researchers responsible for analysis (ENiC & MS).

A reflexive thematic analysis (RTA) method was employed. Braun and Clarke describe reflexive thematic analysis as a theoretically flexible method aimed at “developing, analyzing and interpreting patterns across a qualitative dataset” [21]. This enabled researchers to consider the previous literature and theory (including the initial 2022 study). Unlike research approaches that try to minimise or neutralise the researcher’s influence, reflexive thematic analysis harnesses this influence as a potent analytical tool and enables researchers to use both inductive and deductive processes, semantic or latent interpretation, and realist or constructionist theories [22]. Reflexive thematic analysis allowed researchers to remain self-aware and reflect on their own experiences, biases, and perspectives that directly affect the interpretive process of coding. In our study, we employed a deductive approach. We aimed to explore and analyse evolving stressors related to teacher burnout using the same questions and themes as used in our earlier study [16].

In line with the Braun and Clark RTA method the following steps were utilised (i) immersion and familiarisation; (ii) preliminary coding; (iii) re-reading the data; (iv) developing and re-adjusting themes; (v) clustering themes; (vi) reviewing and selecting quotes as a team to include only pertinent themes and quotes representing the dataset; (vii) writing the report [23].

Responses varied in length from 1 word to 350 words. Although there was no consistent pattern in response length, we found that longer responses tended to focus more on the adverse impact of the pandemic on teacher emotional well-being, their sense of being unsupported or vilified, and the challenges of supporting students’ mental health. In contrast, shorter responses were more frequently associated with workload demands.

The coding process began with an initial reading of the data, resulting in the generation of 75 preliminary codes. These codes were then grouped into broader concepts and categories based on their similarities and were discussed alongside the quotes with the wider research team. The team carefully reviewed the material (raw data and assigned codes), identifying patterns and differences across the quotes and codes, which led to the reduction in the initial 75 codes to 31. The raw data were revisited with these 31 codes, allowing for the identification of sub themes. These were organised further into main and overarching themes at a wider team discussion. Themes were adjusted and refined during these meetings, with a consensus reached on the final analysis. Representative quotes were selected as final examples, which illustrated and validated the themes. The process was iterative, with themes continuously revised until the research team was satisfied that the final analysis captured the essence of the participants’ responses.

ENiC and MS completed these steps independently to minimise bias. The iterative process, with regular reviews and adjustments, ensured the final coding structure was both comprehensive and reflective of the participants’ experiences. A total of four zoom sessions took place with the wider team (EM, ENiC, MS, and FMcN) to discuss the codes and themes. This collaborative process allowed the team to ensure that the final themes and quotes accurately reflected the dataset and were pertinent to the research question.

## 3. Results

### 3.1. Participant Demographics

443 teachers participated, with a mean age of 45.86 (SD: 8.99). The majority were female (*n* = 405, 91.6%), however not all participants answered the free text questions. The response rates to the open-ended questions were as follows: 377 responses to the first question, 280 to the second, and 280 to the third.

### 3.2. Key Themes

75 initial codes were generated which were refined to 31 final codes. These, in turn, were organised into four main themes: (1) Administrative Overload, (2) Unrealistic Expectations, (3) Lack of Community Support and Empathy, and (4) Inadequate Mental Health and Professional Support.

#### 3.2.1. Theme 1: Administrative Overload

The theme of “Administrative Overload” emerged as a major contributor to teacher burnout. Participants expressed feeling overwhelmed by the excessive burden of paperwork, curriculum changes, and managing large class sizes.

Participants described how the continuous increase in administrative tasks diverts attention away from instructional duties noting that the requirement to complete extensive documentation for each student, often for compliance purposes, takes away valuable time that could be spent on lesson planning and student interaction. This, in turn, was highlighted by participants as a contributory factor to reduced job satisfaction and a source of stress. The burden of “paperwork” was the single most common code emerging from the respondents. Respondents underscored how the misalignment between their own expectations of their roles as a teacher and their actual experience leads to frustration and exhaustion. Participants emphasised how teachers are caught in a mire of bureaucracy with multiple direct and indirect adverse consequences, most notably less available time to teach and an increased sense of frustration at the futility of the endless bureaucratic requirements. Participant 81, a self-described experienced teacher of many years, captured this idea well, calling for a “reduction in requirements of [the] Department of Education for unnecessary paperwork which is time consuming and takes from time that I used to spend sourcing new activities and resources 15–20 years ago” (Participant 81).

Another participant pleaded, “Stop bombarding us with new curriculums and programmes without getting the proper training” (Participant 243). Frequent curriculum changes combined with the implementation of new initiatives, when taken together, were recurrent concerns. Teachers expressed immense frustration at having to incorporate and adapt to new teaching initiatives, methodologies, and content, consuming teaching time without the Department of Education providing for practicalities, such as ensuring adequate time for teachers to refine their teaching methods to facilitate successful rollout of these same initiatives. Thus, respondents described being caught in an exasperating loop of ineffectual governance. Respondents outlined how this constant, time-consuming but ineffectual change cycle drains mental energy as teachers are repeatedly required to learn and implement new strategies without effective training or support. Participants signalled a strong desire to see a dramatic reduction in simultaneous initiatives with a focus on prioritisation of effective implementation strategies. Were this implemented, participants anticipated several key benefits, most fundamentally allowing teachers to regain their teaching focus and consequently realigning a perceived professional role with the on-the-job reality. Moreover, participants noted that a secondary consequence of improved strategic prioritisation of key initiatives with adequate resourcing would be a much more effective and successful outcome of these initiatives with improved teacher buy-in.

Finally, the difficulty in managing large class sizes was also noted by participants as a key driver of administrative overload. Respondents described how overloaded classrooms increase the complexity of classroom management and compromises individual student attention, contributing to emotional and physical burnout as teachers struggle to meet the diverse needs of their students.

Participant 133’s response summarily captures the cause and effect of administrative overload which is contributing to burnout in teachers: “There are only so many hours in the day and we are snowed under with paperwork not to mention new initiatives, pilot projects etc” (Participant 133).

#### 3.2.2. Theme 2: Unrealistic Expectations

“Unrealistic Expectations” from parents, the public, and the Department of Education were frequently mentioned as a prominent driver of stress and disillusionment. Teachers reported working in an environment with “massive expectations from parents and society where teachers are expected to be everything” (Participant 154) to a society that demands constant availability and performance.

One particularly important aspect of the unrealistic expectations to which teachers are held was teachers’ frustration with the unfettered access which parents, colleagues, and department figures alike had to them via email, alongside an expectation that they would respond to and conduct meetings outside school hours. Participants noted that this intrusion into personal time leads to stress and undermines work–life balance, as teachers are unable to disconnect and recharge. The lack of boundaries between work and personal life, was identified by participants as a key contributory factor in chronic stress and fatigue, as teachers struggle to meet the endless demands placed on them. One participant described the education system as “having no consideration for personal lives. or that we too are parents” with the pressure of “being on” and available all the time (Participant 383).

Furthermore, participants noted that the lack of consultation and support from the Department of Education results in teachers feeling undervalued and unsupported. This sense of disrespect was exemplified, in the eyes of the participants, by the Department of Education’s ceaseless introduction of poorly resourced initiatives with inadequate training and their imperviousness to teacher feedback in this regard. As one participant simply stated, “all initiatives should have sufficient actual support to make the implementation of same actually doable” (Participant 224). Participants described their perceptions of a top-down and heavy-handed approach from the Department of Education which they noted provided a fertile breeding ground for resentment and a sense of powerlessness, as their professional expertise is devalued. A consideration of the unrealistic expectations which teachers face within society led many respondents to foreground the unreasonable portrayal of teachers in the media.

Participants described how media pressure holds teachers to unrealistic and unreachable standards, eroding their sense of professional capacity and adding to stress. Additionally, participants described how they feel blamed for broader issues beyond their control, which diminishes their motivation and sense of accomplishment. One participant described it as “being vilified by the media for being lazy and uncooperative…we are constantly undermined and feel unvalued and, more insidiously, we cannot raise concerns about these massive issues in the system as it is just portrayed that we are moaning and unwilling rather than unable to meet children’s needs”. This participant concluded their response by stating “it makes me cry on a regular basis” (Participant 154).

Participants emphasised the absolute need for trust in teachers and clear boundaries regarding their roles, advocating for a public campaign to align societal expectations with the realities of the teaching profession. A detailed response from a school principal highlights this point well:

“Clear communication from the Department of Education or M (or Minister Foley) to the parental and greater community re our role in a school [is needed] …. There is a disconnect re what schools must do, and what our role is perceived to be- principals bear the brunt of explaining this. Clear terms and a communication campaign are essential. Schools are not childcare providers, for example. The expectation … makes our job endless, burnout is a result. Schools need clear parameters within which we can work, and the responsibility for communicating same rests with the Department of Education- schools, in particular principals and ancillary staff, should not have to explain what the role is or is not. You wouldn’t bring your math book to the doctor or solicitor or greengrocer” (Participant 22).

#### 3.2.3. Theme 3: Lack of Community Support and Empathy

Participants’ thoughts relating to “Lack of Community Support and Empathy” underscored the fundamental role of true community within the school system. A truly effective school community mirrors the foundational requirements of all functional communities; those participants identified societal support and understanding as critical. Participants—who, as discussed, already feel overloaded by administrative work and disillusioned by unrealistic expectations—frequently report a distinct lack of empathy and understanding from the communities they serve and the employers they work for. They report a sense of disconnect, working as one participant put it in a “very isolating profession without the collaboration and camaraderie that can make challenging work more manageable”. (Participant 294). As another put it, “To be honest, supporting staff has never been a priority for schools and teachers are gaslit into believing that all we need to do is more training” to improve their teaching experiences (Participant 68).

Emerging from and related to this finding, participants frequently expressed a desire for greater respect, empathy, and kindness from the Department of Education, management, media, parents, and the public at large. Calls for greater “respect” accounted for one of the most frequently recurrent codes and underscores the moral dimension of the teacher burnout issue. One participant captured this sentiment well: “Be a little more understanding and less demanding on a human level A little empathy would go a long way Kindness from principal would be welcome- hire principals who are skilled in dealing with people, not paper. Fewer obnoxious Aladdin messages containing threats and nasty tones.” (Participant 107).

A separate but equally important concept captured within this theme is the participants’ recurrent advocacy and concern for students with special educational needs (as distinct from mental health needs, which is covered below, and to the extent that this is possible). Participants very frequently decry the difficulty in accessing specialised support for these students and describe a foreboding sense of responsibility to compensate for these gaps. As one participant stated, “teachers are expected to be everything to the children in their care—teacher, parent, SLT [Speech and Language Therapist], psychologist, OT [Occupational Therapist], etc., and to provide these services whilst teaching a full curriculum to 30 children with diverse needs”. (Participant 154).

Finally, participants called for collaborative policy development involving the whole community to foster respect, empathy, and care. By building a supportive school environment where teachers, students, parents, and community members work together, burnout can be reduced through shared responsibility and understanding. This approach promotes a sense of belonging and purpose, enhancing teacher resilience and satisfaction.

#### 3.2.4. Theme 4: Inadequate Mental Health and Professional Support

The theme “Inadequate Mental Health and Professional Support” underscored the need for accessible mental health resources and support for teachers and students alike.

On the one hand, participants frequently report an awareness of an emerging youth mental health crisis, the fact that “anxiety and mental health issues in children and their families have massively increased” as one participant outlined (Participant 306). (Several participants connect this phenomenon to the unique ways in which young people were affected by the pandemic). Furthermore, participants frequently describe the difficulty young people have in accessing professional mental health supports and a sense of responsibility to “plug the gaps”, similar to the discussion above regarding students with special educational needs. Teachers are essentially having to act beyond the scope of their practice and carry risks and responsibilities that rightfully should be diffused among healthcare professionals. The effect of this, unsurprisingly, is emotional exhaustion, a core feature of burnout: “We are expected to be there to support the pupils and give from our cups, but we cannot do that if we in turn are not supported, and our cups are empty”. (Participant 16).

On the other hand, then, participants highlight their own unmet psychological needs while frequently taking a dim view on the institutional efforts to promote mental health: “well-being for teachers is just lip service … does not take into account how emotionally demanding teaching has become due largely to the anxiety in children and learning/behavioural/social skills deficits in children caused largely by the pandemic” (Participant 256). Participants also articulate a range of suggestions to help address this issue, from formalised peer support networks, bolstered in-house counselling services, and sponsored external support from mental health professionals.

Taken as a whole, what emerges is a vicious circle: teachers’ caring for students with worsening mental health are themselves suffering with worsening mental health, in some part due to this added responsibility. Both parties in turn struggle to access mental health support, and the cycle continues.

## 4. Discussion

This study explored the factors contributing to teacher burnout in post-COVID-19 Ireland, highlighting four themes: administrative overload, unrealistic expectations, lack of community support and empathy, and inadequate mental health and professional support. The findings build on and align with existing research, particularly the studies led by our colleagues Minihan et al., which investigated occupational stress in Irish teachers during the COVID-19 pandemic (2022). As with that earlier work, ours was a mixed methods study which produced both the qualitative data presented here, as well as quantitative data published elsewhere, which drew on the Copenhagen Burnout Index (CBI) [15,16]. In terms of the qualitative data, both studies highlight administrative overload as a significant factor in teacher stress. However, while Minihan et al. focused on the immediate challenges posed by the pandemic, such as transitioning to online teaching and managing health risks, this current study reveals that administrative burdens have persisted beyond the pandemic.

Other studies have found a lack of abatement in stress for teachers post-pandemic. A study by Baker and Koedel among teachers in USA found that working conditions showed a prolonged deterioration that persisted after the peak of the pandemic. They found no difference in terms of socioeconomic status; however, schools that had adapted more online learning showed a larger decline in conditions. Working conditions post-pandemic were unrelated to socioeconomic disadvantage but were poorer in schools where online learning was the predominant mode of teaching during the 2020–2021 [18]. This highlights the need to ensure that teachers are adequately trained and supported when moving towards more standard hybrid models. Given the realisation that stress had already existed pre-COVID-19 as one of the main reasons for teacher attrition, even more so than pay [24] if unaddressed, could lead to a mass exodus from an already denied service.

Furthermore, unrealistic expectations were identified as adversely impacting both cohorts of teachers. Minihan et al. described the pressure on teachers to adapt rapidly to remote teaching and maintain educational standards despite technological and pedagogical challenges [15]. The current study extends these findings by showing that these expectations have continued, with teachers feeling pressured to be constantly available and to implement new initiatives without adequate support. These concerns mirror similar observations across a range of professional groups both in the public and private sector, pointing to an urgent need for innovative strategies to address this growing stressor [25,26]. Realistic boundaries and expectations are a prerequisite to teacher well-being. Within this context, it is essential to consider the need to develop frameworks regarding the evolving “always on” professional culture across a myriad of occupations. Clear policies need to be drawn up as to the use of email by parents to contact teachers directly and the expectation of immediate availability. This is best shaped within a broader societal conversation to garner true engagement.

Moreover, the lack of community support and empathy noted in both studies, is also reflective of larger societal trends. Many people-facing professions (such as police and health professionals) report increasing demands with decreased community respect and support [27]. Indeed, more broadly, consistent data globally demonstrates a dramatic fall-off in societal trust of government institutions/agencies and those in positions of authority or power [27]. At a micro-level, elements of the changed parental stance towards teachers may in part reflect this larger societal trend. Both studies identified inadequate mental health support as a critical issue, with Minihan et al. noting the increased stress and anxiety levels among teachers during the pandemic [15].

Our findings further suggest that the lack of accessible mental health resources remains a significant concern. Teachers continue to face high levels of stress without sufficient support systems, highlighting the urgent need for improved mental health services and professional development opportunities. However, it is imperative that the provision of well-being and or mental health support to alleviate occupational stress are not merely “bolt on” provisions. Causal stressors must be addressed contemporaneously.

Our four themes align with the JD-R model proposed by Bakker and Demerouti [6], which suggests that burnout results when job demands outweigh the resources available to employees. In our study, teachers reported high job demands (such as administrative overload and unrealistic expectations) alongside insufficient resources (lack of support, mental health services, and community empathy) setting fertile ground for burnout. Our themes may also be interpretated using an earlier model by Maslach and Leiter [28]. This model identifies six domains of workload, control, reward, community, fairness, and values, as crucial factors in understanding the causes of burnout. The authors emphasise how imbalances in any of these domains may lead to chronic stress and emotional exhaustion in employees. With reference to this model, the theme of Administrative Overload suggests an imbalance in the domain of “workload”. The increasing demands of paperwork, curriculum changes, and large class sizes, referred to by teachers, may contribute to emotional exhaustion and stress. Similarly, the theme of Unrealistic Expectations links closely to that of “control”. In our study, teachers report feeling a lack of autonomy in their roles, particularly when they are expected to meet constant demands from parents and administrators, with little flexibility or agency. The theme of Lack of Community Support and Empathy fits within the domain of “community”, where teachers experience a sense of isolation and a lack of understanding from their colleagues and employers, undermining the supportive networks that are essential for mitigating burnout. Lastly, the theme of Inadequate Mental Health Support aligns with the domains of “reward” and “fairness”. Teachers perceive a lack of adequate resources and support for their own mental health, highlighting the imbalance between the demands placed on them and the insufficient support they receive, leading to feelings of inequity and diminishing their sense of personal accomplishment. Both models highlight a scenario where the demands (workload, expectations) outweigh the resources (control, rewards, support, fairness), creating a fertile ground for burnout.

Addressing these interconnected themes through systemic changes—such as reducing administrative burdens, recalibrating expectations, fostering empathy, and expanding mental health resources—can help balance demands and resources, mitigating teacher burnout and supporting a healthier educational environment. The need for such acroscopic and systemic change is clearly articulated in the microscopic and individual voices of this study. Multiple respondents identified a sense of “lip service” being paid to occupational health and well-being, without evidence of meaningful, sustained or resourced efforts to mitigate against burnout. Among other things, respondents called for regular in-service days for promoting staff well-being and team-building, and resourcing staff access to external mental health services, and fostering a culture

The findings of this study have several implications for policy and practice in education. First, reducing administrative burdens and streamlining processes can help alleviate stress and allow teachers to focus on their primary roles as educators. Policymakers should consider implementing measures to simplify administrative tasks and ensure that curriculum changes are accompanied by adequate training and resources. Within this, the voices of teachers must be sought upon, listened to and respected as is the case in any well-performing organisation.

Secondly, establishing clear boundaries regarding teachers’ roles and responsibilities can help manage unrealistic expectations. This is best considered within a broader governmental framework as to the need to target the dominance of tech-based demands in out-of-hour contexts. Schools and policymakers should work together to create policies that protect teachers’ personal time and promote a healthy work–life balance. Public campaigns to raise awareness about the realities of the teaching profession can also help align societal expectations with teachers’ actual roles.

Third, fostering a supportive school community that prioritises empathy, and collaboration is essential for reducing burnout. Schools should encourage open communication and mutual support among teachers, students, parents, and community members. Collaborative policy development involving all stakeholders can help build a sense of shared responsibility and respect, enhancing teacher resilience and satisfaction.

Finally, improving mental health and professional support for teachers is crucial for addressing burnout. Schools and policymakers should prioritise funding for accessible mental health resources and professional development opportunities. Providing teachers with the necessary training and support to manage student mental health issues can also alleviate stress and improve overall well-being.

This study highlights the necessity of addressing both pre-existing and pandemic-exposed stressors to optimise interventions for teacher burnout. Effective strategies must simultaneously reduce job demands and enhance available resources while recognising the unique and individualised nature of stressors and their varying relationships with different dimensions of burnout [6]. A scoping review examining burnout interventions for teachers between 2018 and 2022 identified 16 different stress-reduction approaches across 40 worldwide studies [29]. These included Mindfulness-Based Interventions (MBIs), Cognitive Behavioural Therapy (CBT), yoga, Rational Emotive Behavioural Therapy (REBT among others and showed promise. Given the significant impact of burnout on both educators and students, which do not seem to be abating post COVID-19, school-based interventions should be prioritised to support teachers’ well-being and resilience [29].

## 5. Limitations

This study has several limitations that should be noted. First, while the methodology mirrors the approach used in a previous study [16], the cohort of teachers in the current study is different, meaning the findings can only reflect group-level changes, not individual-level comparisons across the two time points. Additionally, we were unable to link any teachers’ experiences with specific, documented changes within their schools which may have affected their experiences.

Moreover, due to the anonymous nature of the research, it was not possible to determine the response rate from each recruitment stream (INTO, ILSA, and School Invitations), nor to assess the proportion of responses from different school levels, such as primary versus post-primary, or geographical spread. This lack of demographic linkage means the representativeness of the sample within the broader Irish teaching population remains uncertain. The sample size is also relatively small compared to the overall teaching population in Ireland. Despite these limitations, the qualitative approach offers valuable insights into teachers’ personal perceptions of stress, burnout, and their recommendations for stress reduction moving forward.

A key strength of this study is its focus on lived experiences, providing authentic insights into teachers’ personal perceptions of stress and burnout in Ireland. By capturing teachers’ voices directly, the study highlights the nuanced realities of their day-to-day challenges, offering a deep understanding of how they experience and respond to ongoing pressures during and after the pandemic. The themes identified—such as administrative overload, unrealistic expectations, lack of support, and inadequate mental health resources—are critical in understanding the root causes of burnout and help broaden the conceptual framework of the JD-R model, viewing each as single latent dimension. Addressing these themes is essential for reducing teacher stress and fostering a more supportive and sustainable work environment, which can, in turn, improve both teacher well-being and the overall educational experience.

## 6. Conclusions

This study highlights the ongoing challenges faced by teachers in post-COVID-19 Ireland, emphasising the urgent need for systemic changes to address burnout. The findings identify critical factors contributing to teacher stress, such as administrative overload, unrealistic expectations, lack of societal support, and inadequate mental health resources. These themes not only align with but also extend previous research, including the work of Minihan et al. [15,16], which highlights the continuing impact of COVID-19 on teachers and the persistence of these stressors beyond the pandemic.

The themes identified in this study map onto established frameworks such as the Job Demand–Resource (JD-R) model [5] and Maslach’s six domains of work life [28], revealing how an imbalance between job demands and available resources creates fertile ground for burnout. To foster a more supportive and sustainable educational environment, it is vital to reduce administrative burdens, recalibrate unrealistic expectations, promote societal empathy, and expand mental health support. While individual interventions can help build teacher resilience, they must be accompanied by changes in organisational culture and practical systems. The findings underscore the necessity of comprehensive support systems that not only address immediate stressors but also promote long-term teacher well-being. Addressing these interconnected themes is crucial for creating a healthier educational environment that benefits both teachers and students alike.

## Data Availability

The data presented in this study are available on request from the corresponding author due to privacy reasons.
